# On Generalized Difference Hahn Sequence Spaces

**DOI:** 10.1155/2014/398203

**Published:** 2014-05-13

**Authors:** Kuldip Raj, Adem Kiliçman

**Affiliations:** ^1^School of Mathematics, Shri Mata Vaishno Devi University, Katra 182320, India; ^2^Department of Mathematics and Institute for Mathematical Research, University Putra Malaysia (UPM), 43400 Serdang, Selangor, Malaysia

## Abstract

We construct some generalized difference Hahn sequence spaces by mean of sequence of modulus functions. The topological properties and some inclusion relations of spaces *h*_*p*_(*ℱ*, *u*, Δ^*r*^) are investigated. Also we compute the dual of these spaces, and some matrix transformations are characterized.

## 1. Introduction and Preliminaries

By a sequence space, we understand a linear subspace of the space *w* = *ℂ* ^*ℕ*^ of all real or complex-valued sequences, where *ℂ* denotes the complex field and *ℕ* = 0,1, 2,… . For *x* = (*x*_*k*_) ∈ *w*, we write *l*_*∞*_, *c*, and *c*_0_ for the classical spaces of all bounded, convergent, and null sequences, respectively. Also by *bs*, *cs*, and *l*_*p*_ we denote the space of all bounded, convergent, and *p*-absolutely convergent series, which are Banach spaces with the following norms: ||*x*||_*bs*_ = ||*x*||_*cs*_ = sup⁡_*n*_|∑_*k*=1_^*n*^ *x*_*k*_| and ||*x*||_*l*_*p*__ = (∑_*k*_ | *x*_*k*_|^*p*^)^1/*p*^, respectively. Additionally, the spaces *bv* ^*p*^ and ∫*λ* are defined by

(1) bvp={x=(xk)∈w:∑k=1∞|xk−xk−1|p<∞},∫λ={x=(xk)∈w:(kxk)∈λ}.

A coordinate space (or a *K*-space) is a vector space of numerical sequences, where addition and scalar multiplication are defined pointwise. That is, a sequence space *λ* with a linear topology is called a *K*-space provided that each of the maps *p*_*i*_ : *λ* → *ℂ* defined by *p*_*i*_(*x*) = *x*_*i*_ is continuous for all *i* ∈ *ℕ*. A *BK*-space is a *K*-space, which is also a Banach space with continuous coordinate functionals *f*_*k*_(*x*) = *x*_*k*_, (*k* = 1,2,…). A *K*-space *λ* is called an *FK*-space provided that *λ* is a complete linear metric space. An *FK*-space whose topology is normable is called a *BK*-space. If a normed sequence space *λ* contains a sequence (*b*_*n*_) with the property that for every *x* ∈ *λ* there is a unique sequence of scalars (*α*_*n*_) such that

(2) $$\begin{array}{c} \underset{n\to \infty }{\lim}||x-\left(\alpha_{0}b_{0}+\alpha_{1}b_{1}+\cdots +\alpha_{n}b_{n}\right)||=0, \end{array}$$

then (*b*_*n*_) is called Schauder basis (or briefly basis) for *λ*. The series ∑*α*_*k*_ *b*_*k*_ which has the sum *x* is then called the expansion of *x* with respect to (*b*_*n*_) and written as *x* = ∑*α*_*k*_ *b*_*k*_. An *FK*-space *λ* is said to have *AK* property, if *ϕ* ⊂ *λ* and {*e* ^*k*^} is a basis for *λ*, where *e* ^*k*^ is a sequence whose only nonzero term is 1 in *k*th place for each *k* ∈ *ℕ* and *ϕ* = span⁡{*e* ^*k*^}, the set of all finitely nonzero sequences. If *ϕ* is dense in *λ*, then *λ* is called an *AD*-space, and thus *AK* implies *AD*.

The notion of difference sequence spaces was introduced by Kizmaz [1], who defined the sequence spaces as follows:

(3) Z(Δ)={x=(xk)∈w:(Δxk)∈Z}for  Z=c,c0,l∞,

where Δ*x* = (Δ*x*_*k*_) = (*x*_*k*_ − *x*_*k*+1_). The notion was further generalized by Et and Çolak [2] by introducing the spaces. Let *r* be a nonnegative integer; then,

(4) Z(Δr)={x=(xk)∈w:(Δrxk)∈Z}for  Z=c,c0,l∞,

where Δ^*r*^ *x* = (Δ^*r*^ *x*_*k*_) = (Δ^*r*−1^ *x*_*k*_ − Δ^*r*−1^ *x*_*k*+1_) and Δ^0^ *x*_*k*_ = *x*_*k*_ for all *k* ∈ *ℕ*. The generalized difference sequence has the following binomial representation:

(5) $$\begin{array}{c} \Delta^{r}x_{k}=\overset{r}{\underset{m=0}{\sum }}{\left(-1\right)}^{m}\left(\begin{array}{c} r\\ m \end{array}\right)x_{k+m}. \end{array}$$

Later concept have been studied by Bektaş et al. [3] and Et and Esi [4]. Another type of generalization of the difference sequence spaces is due to Tripathy and Esi [5] who studied the spaces *l*_*∞*_(Δ_*v*_), *c*(Δ_*v*_), and *c*_0_(Δ_*v*_). Recently, Esi et al. [6] and Tripathy et al. [7] have introduced a new type of generalized difference operators and unified those as follows.

Let *r*, *v* be nonnegative integers; then, for *Z* a given sequence space, we have

(6) $$\begin{array}{c} Z\left(\Delta_{v}^{r}\right)=\left\{x=\left(x_{k}\right)\in w:\left(\Delta_{v}^{r}x_{k}\right)\in Z\right\} \end{array}$$

for *Z* = *c*, *c*_0_ and *l*_*∞*_, where Δ_*v*_^*r*^ *x* = (Δ_*v*_^*r*^ *x*_*k*_) = (Δ_*v*_^*r*−1^ *x*_*k*_ − Δ_*v*_^*r*−1^ *x*_*k*+*v*_) and Δ_*v*_^0^ *x*_*k*_ = *x*_*k*_ for all *k* ∈ *ℕ*.

Let *X* be a linear metric space. A function *p* : *X* → ℝ is called paranorm, if

1. *p*(*x*) ≥ 0 for all *x* ∈ *X*,
2. *p*(−*x*) = *p*(*x*) for all *x* ∈ *X*,
3. *p*(*x* + *y*) ≤ *p*(*x*) + *p*(*y*) for all *x*, *y* ∈ *X*,
4. (*λ*_*n*_) is a sequence of scalars with *λ*_*n*_ → *λ* as *n* → *∞* and (*x*_*n*_) is a sequence of vectors with *p*(*x*_*n*_ − *x*) → 0 as *n* → *∞*, then *p*(*λ*_*n*_ *x*_*n*_ − *λx*) → 0 as *n* → *∞*.

A paranorm *p* for which *p*(*x*) = 0 implies *x* = 0 is called total paranorm and the pair (*X*, *p*) is called a total paranormed space. It is well known that the metric of any linear metric space is given by some total paranorm (see [8], Theorem 10.4.2, pp. 183). For more details about sequence spaces (see [9, 10]) and the references therein.

A modulus function is a function *f* : [0, *∞*)→[0, *∞*) such that

1. *f*(*x*) = 0 if and only if *x* = 0,
2. *f*(*x* + *y*) ≤ *f*(*x*) + *f*(*y*), for all *x*, *y* ≥ 0,
3. *f* is increasing,
4. *f* is continuous from the right at 0.

It follows that *f* must be continuous everywhere on [0, *∞*). The modulus function may be bounded or unbounded. For example, if we take *f*(*x*) = *x*/(*x* + 1), then *f*(*x*) is bounded. If *f*(*x*) = *x* ^*p*^, 0 < *p* < 1, then the modulus function *f*(*x*) is unbounded. Subsequentially, modulus function has been discussed in ([11–14]) and references therein.

Let *λ* and *μ* be two sequence spaces and *A* = (*a*_*nk*_) be an infinite matrix of complex numbers, where *k*, *n* ∈ *ℕ*. Then, we say that *A* defines a matrix mapping from *λ* into *μ*, and we denote it by writing *A* : *λ* → *μ* for every sequence *x* = (*x*_*k*_) ∈ *λ*. The sequence *Ax* = {(*Ax*)_*n*_}, the *A*-transform of *x*, is in *μ*, where

(7) $$\begin{array}{c} {\left(Ax\right)}_{n}=\underset{k}{\sum }a_{nk}x_{k}\;\text{for each}\;\;n\in \mathbb{N}. \end{array}$$

For simplicity in notation, here and in what follows, the summation without limits runs from 0 to *∞*. By (*λ* : *μ*), we denote the class of all matrices *A* such that *A* : *λ* → *μ*. Thus, *A* ∈ (*λ* : *μ*) if and only if the series on the right side of (7) converges for each *n* ∈ *ℕ* and each *x* ∈ *λ* and we have *Ax* = {(*Ax*)_*n*_}_*n*∈*ℕ*_ ∈ *μ* for all *x* ∈ *λ*. A sequence *x* is said to be *A*-summable to *l* if *Ax* converges to *l* which is called the *A*-limit of *x*.

The matrix domain *λ*_*A*_ of an infinite matrix *A* in a sequence space *λ* is defined by

(8) $$\begin{array}{c} \lambda_{A}=\left\{x=\left(x_{k}\right)\in w:Ax\in \lambda \right\} \end{array}$$

which is a sequence space (for several examples of matrix domains, see [15] p. 49–176). In [16], Başar and Altay have defined the sequence space *bv*_*p*_ which consists of all sequences such that Δ-transforms of them are in *l*_*p*_, where Δ denotes the matrix Δ = (*δ*_*nk*_) as follows:

(9) $$\begin{array}{c} \Delta =\delta_{nk}=\{\begin{array}{cc} {\left(-1\right)}^{n-k}, & \left(n-1\leq k\leq n\right);\\ 0, & \left(0\leq k<n-1\;\text{or}\;k>n\right) \end{array} \end{array}$$

for all *k*, *n* ∈ *ℕ*. The space [*l*(*p*)]_*A*^*u*^_ = *bv*(*u*, *p*) has been studied by Başar et al. [17], where

(10) $$\begin{array}{c} A^{u}=a_{nk}^{u}=\{\begin{array}{cc} {\left(-1\right)}^{n-k}u_{k}, & \left(n-1\leq k\leq n\right);\\ 0, & \left(0\leq k<n-1\;\text{or}\;k>n\right). \end{array} \end{array}$$

Hahn [18] introduced the *BK*-space *h* of all sequences *x* = (*x*_*k*_) such that

(11) $$\begin{array}{c} h=\left\{x:\overset{\infty }{\underset{k=1}{\sum }}k\left|\Delta x_{k}\right|<\infty ,\;\;\underset{k\to \infty }{\lim}x_{k}=0\right\}, \end{array}$$

where Δ*x*_*k*_ = *x*_*k*_ − *x*_*k*+1_, for all *k* ∈ *ℕ*. The following norm:

(12) $$\begin{array}{c} {||x||}_{h}=\underset{k}{\sum }k\left|\Delta x_{k}\right|+\underset{k}{\sup}\left|x_{k}\right| \end{array}$$

was defined on the space *h* by Hahn [18] (and also [19]). Rao ([20], Proposition 2.1) defined a new norm on *h* as ||*x*|| = ∑_*k*_ *k* | Δ*x*_*k*_|. G. Goes and S. Goes [19] proved that the space *h* is a *BK*-space.

Hahn proved the following properties of the space *h*.

**Lemma 1 .**

(i) *h* is a Banach space.

(ii) *h* ⊂ *l*_1_∩∫*c*_0_.

(iii) *h* ^*β*^ = *σ*_*∞*_.

In [19], G. Goes and S. Goes studied functional analytic properties of the *BK*-space *bv*_0_∩*dl*_1_. Additionally, G. Goes and S. Goes considered the arithmetic means of sequences in *bv*_0_ and *bv*_0_∩*dl*_1_ and used an important fact which the sequence of arithmetic means (*n* ^−1^∑_*k*=1_^*n*^ *x*_*k*_) of *x* ∈ *bv*_0_ is a quasiconvex null sequence. And also G. Goes and S. Goes proved that *h* = *l*_1_∩∫*bv* = *l*_1_∩∫*bv*_0_.

Rao [20] studied some geometric properties of Hahn sequence space and gave the characterizations of some classes of matrix transformations. Balasubramanian and Pandiarani [21] defined the new sequence space *h*(*F*) called the Hahn sequence space of fuzzy numbers and proved that *β* and *γ* duals of *h*(*F*) is the Cesàro space of the set of all fuzzy bounded sequences. Kirişci [22] compiled to studies on Hahn sequence space and defined a new Hahn sequence space by Cesàro mean in [23].

In [24], Kirişci introduce the sequence space *h*_*p*_ by

(13) $$\begin{array}{c} h_{p}=\left\{x:\overset{\infty }{\underset{k=1}{\sum }}k{\left|\Delta x_{k}\right|}^{p}<\infty ,\underset{k\to \infty }{\lim}x_{k}=0\right\},\;\left(1<p<\infty \right), \end{array}$$

where Δ*x*_*k*_ = *x*_*k*_ − *x*_*k*+1_, for all *k* ∈ *ℕ*. If we take *p* = 1, *h*_*p*_ = *h* which are called Hahn sequence spaces. We denote the collection of all finite subsets of *ℕ* by *F*.

Let *ℱ* = (*f*_*k*_) be a sequence of modulus functions, *p* = (*p*_*k*_) be a bounded sequence of positive real numbers, and *u* = (*u*_*k*_) be a sequence of strictly positive real numbers. In the present paper we defined the following sequence space:

(14) hp(ℱ,u,Δr)  ={x:∑k=1∞fk[(k|ukΔrxk|)pk]<∞,  lim⁡k→∞xk=0},(1<p<∞),

where Δ^*r*^ *x* = (Δ^*r*^ *x*_*k*_) = (Δ^*r*−1^ *x*_*k*_ − Δ^*r*−1^ *x*_*k*+1_) and Δ^0^ *x*_*k*_ = *x*_*k*_ for all *k* ∈ *ℕ*. Define the sequence *y* = (*y*_*k*_), which will be frequently used, by *M*-transform of a sequence *x* = (*x*_*k*_); that is,

(15) $$\begin{array}{c} y_{k}={\left(Mx\right)}_{k}=f_{k}u_{k}k\left(\Delta^{r-1}x_{k}-\Delta^{r-1}x_{k+1}\right), \end{array}$$

where *M* = (*m*_*nk*_) with

(16) $$\begin{array}{c} m_{nk}=\{\begin{array}{cc} n, & \left(n=k\right);\\ -n, & \left(n+1=k\right);\\ 0, & \text{other} \end{array} \end{array}$$

for all *k*, *n* ∈ *ℕ*.

If we take *u* = (*u*_*k*_) = 1, *r* = 1 and *ℱ* = *f*_*k*_(*x*) = *x* for all *k* ∈ *ℕ*, then we get the sequence space *h*_*p*_ defined by [24] Kirişci. By taking *u* = (*u*_*k*_) = 1, *r* = 1,  *ℱ* = *f*_*k*_(*x*) = *x*, and *p* = (*p*_*k*_) = 1 for all *k* ∈ *ℕ*, we obtained a Hahn sequence space defined by Hanh [18].

The following inequality will be used throughout the paper.

Let *p* = (*p*_*k*_) be a sequence of positive real numbers with 0 < *p*_*k*_ ≤ sup⁡_*k*_ *p*_*k*_ = *H*, and let *K* = max⁡{1, 2^*H*−1^}. Then, for the factorable sequences (*a*_*k*_) and (*b*_*k*_) in the complex plane, we have

(17) $$\begin{array}{c} {\left|a_{k}+b_{k}\right|}^{p_{k}}\leq K\left({\left|a_{k}\right|}^{p_{k}}+{\left|b_{k}\right|}^{p_{k}}\right). \end{array}$$

The main purpose of this paper is to study some difference Hahn sequence spaces by mean of sequence of modulus functions. We will study some topological and algebraic properties of the sequence spaces *h*_*p*_(*ℱ*, *u*, Δ^*r*^) in Section 2. In Section 3 we will determine the *α*-, *β*-, and *γ*-duals of the spaces *h*_*p*_(*ℱ*, *u*, Δ^*r*^). Finally, we also made an attempt to characterize some matrix transformations on the spaces *h*_*p*_(*ℱ*, *u*, Δ^*r*^).

## 2. Main Results

The purpose of this section is to study the properties like linearity, paranorm, and relevant inclusion relations in the spaces *h*_*p*_(*ℱ*, *u*, Δ^*r*^).

**Theorem 2 .**

The sequence space *h*_*p*_(*ℱ*, *u*, Δ^*r*^) is a linear space over the complex field *ℂ*.

**Proof**

Let *x* = (*x*_*k*_), *y* = (*y*_*k*_) ∈ *h*_*p*_(*ℱ*, *u*, Δ^*r*^) and *ρ*, *ϱ* ∈ *ℂ*. Then their exist integers *M*_*ρ*_ and *N*_*ϱ*_ such that |*ρ* | ≤*M*_*ρ*_ and |*ϱ* | ≤*N*_*ϱ*_. By using the inequality (17) and the properties of modulus function, we have

(18) ∑k=1∞fk[(k|ukΔr(ρxk+ϱyk)|)pk]  ≤∑k=1∞fk[(k|uk(ρΔrxk+ϱΔryk)|)pk]  ≤K∑k=1∞fk[Mρ(k|ukΔrxk|)pk]   +K∑k=1∞fk[Nϱ(k|ukΔryk|)pk]  ≤KMρH∑k=1∞fk[(k|ukΔrxk|)pk]   +KNϱH∑k=1∞fk[(k|ukΔryk|)pk]<∞.

Thus, *ρx* + *ϱy* ∈ *h*_*p*_(*ℱ*, *u*, Δ^*r*^). This proves that *h*_*p*_(*ℱ*, *u*, Δ^*r*^) is a linear space over the field of complex number *ℂ*.

**Theorem 3 .**

Let *ℱ* = (*f*_*k*_) be a sequence of modulus functions and *u* = (*u*_*k*_) be any sequence of strictly positive real numbers. Then *h*_*p*_(*ℱ*, *u*, Δ^*r*^) is a paranormed space with the paranorm defined by

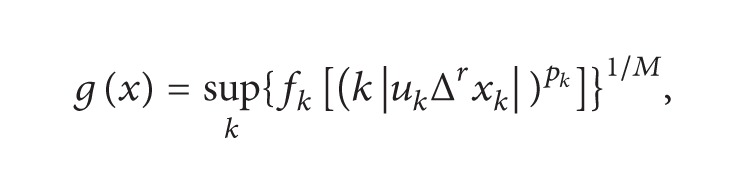
(19)
where *H* = sup⁡_*k*_ *p*_*k*_ < *∞* and *M* = max⁡(1, *H*).

**Proof**

Clearly *g*(*x*) = *g*(−*x*) for all *x* ∈ *h*_*p*_(*ℱ*, *u*, Δ^*r*^). It is trivial that *u*_*k*_Δ^*r*^ *x*_*k*_ = 0 for *x* = 0. Since *p*_*k*_/*M* ≤ 1 using Minkowski's inequality, we have

(20) {fk[(k|ukΔr(xk+yk)|)pk]}1/M  ≤{fk[(k|(ukΔrxk+ukΔryk)|)pk]}1/M  ≤{fk[(k|ukΔrxk|)pk]}1/M+{fk[(k|ukΔryk|)pk]}1/M.

Hence *g*(*x* + *y*) ≤ *g*(*x*) + *g*(*y*). Finally to check the continuity of scalar multiplication, let us take a complex number *δ* by definition, we have

(21) $$\begin{array}{c} g\left(\delta x\right)=\underset{k}{\sup}{\left\{f_{k}\left[{\left(k\left|u_{k}\Delta^{r}\delta x_{k}\right|\right)}^{p_{k}}\right]\right\}}^{1/M}\leq K_{\delta }^{H/M}g\left(x\right), \end{array}$$

where *K*_*δ*_ is a positive integer such that |*δ* | ≤*K*_*δ*_. Let *δ* → 0 for any fixed *x* with *g*(*x*) = 0. By definition for |*δ* | <1, we have

(22) $$\begin{array}{c} \underset{k}{\sup}{\left\{f_{k}\left[{\left(k\left|u_{k}\Delta^{r}x_{k}\right|\right)}^{p_{k}}\right]\right\}}^{1/M}<\epsilon \;\text{for}\;\;n>N\left(\epsilon \right). \end{array}$$

Also for 1 ≤ *n* ≤ *N*, taking *δ* small enough, since *f*_*k*_ is continuous for each *k*, we have

(23) $$\begin{array}{c} \underset{k}{\sup}{\left\{f_{k}\left[{\left(k\left|u_{k}\Delta^{r}x_{k}\right|\right)}^{p_{k}}\right]\right\}}^{1/M}<\epsilon . \end{array}$$

Equations (22) and (23) imply that *g*(*δx*) → 0 as *δ* → 0. This completes the proof.

**Theorem 4 .**

Let *ℱ* = (*f*_*k*_) be a sequence of modulus functions and *ϕ* = lim⁡_*t*→*∞*_(*f*_*k*_(*t*)/*t*) > 0. Then *h*_*p*_(*ℱ*, *u*, Δ^*r*^) ⊂ *h*_*p*_(*u*, Δ^*r*^).

**Proof**

Let *ϕ* > 0. By definition of *ϕ*, we have *f*_*k*_(*t*) ≥ *ϕ* · *t*, for all *t* ≥ 0. Since *ϕ* > 0, we have *t* ≤ (1/*ϕ*)*f*_*k*_(*t*) for all *t* ≥ 0. Let *x* = (*x*_*k*_) ∈ *h*_*p*_(*ℱ*, *u*, Δ^*r*^). Thus, we have

(24) ∑k=1∞[(k|ukΔrxk|)pk]≤1ϕ∑k=1∞fk[(k|ukΔrxk|)pk]<∞.

Which implies that *x* = (*x*_*k*_) ∈ *h*_*p*_(*u*, Δ^*r*^). This completes the proof.

**Theorem 5 .**

*h*_*p*_(*ℱ*, *u*, Δ^*r*^) = *l*_*p*_(*ℱ*, *u*, Δ^*r*^)∩∫*bv* ^*p*^(*ℱ*, *u*, Δ^*r*^) = *l*_*p*_(*ℱ*, *u*, Δ^*r*^)∩∫*bv*_0_^*p*^(*ℱ*, *u*, Δ^*r*^).

**Proof**

We consider

(25) $$\begin{array}{c} f_{k}u_{k}\left(k\Delta^{r}x_{k}\right)\leq f_{k}u_{k}\left(x_{k}\right)+f_{k}u_{k}\left(\Delta^{r}\left(kx_{k}\right)\right). \end{array}$$

Then, for *x* ∈ *l*_*p*_(*ℱ*, *u*, Δ^*r*^)∩∫*bv* ^*p*^(*ℱ*, *u*, Δ^*r*^),

(26) $$\begin{array}{c} \overset{n}{\underset{k=1}{\sum }}f_{k}u_{k}\left(k\left|\Delta^{r}x_{k}\right|\right)\leq \overset{n}{\underset{k=1}{\sum }}f_{k}u_{k}\left|x_{k}\right|+\overset{n}{\underset{k=1}{\sum }}f_{k}u_{k}\left|\Delta^{r}\left(kx_{k}\right)\right|, \end{array}$$

and from |*a* + *b*|^*p*_*k*_^ ≤ 2^*p*_*k*_^(|*a*|^*p*_*k*_^ + |*b*|^*p*_*k*_^), (1 ≤ *p*_*k*_ < *∞*), we obtain

(27) ∑k=1nfkuk[kpk|Δrxk|pk]  ≤2pkfkuk[∑k=1n|xk|pk+∑k=1n|Δr(kxk)|pk].

For each positive integer *s*, we get

(28) ∑k=1sfkuk[kpkΔrxk|pk]  ≤2pkfkuk[∑k=1s|xk|pk+∑k=1s|Δrkxk|pk],

and as *s* → *∞*,

(29) ∑k=1∞fkuk[kpk|Δrxk|pk]  ≤2pkfkuk[∑k=1∞|xk|pk+∑k=1∞|Δr(kxk)|pk]

and lim⁡_*k*→*∞*_ *x*_*k*_ = 0. Then *x* ∈ *h*_*p*_(*ℱ*, *u*, Δ^*r*^) and

(30) $$\begin{array}{c} l_{p}\left(ℱ,u,\Delta^{r}\right)\cap \int bv^{p}\left(ℱ,u,\Delta^{r}\right)\subset h_{p}\left(ℱ,u,\Delta^{r}\right). \end{array}$$

Let *x* ∈ *h*_*p*_(*ℱ*, *u*, Δ^*r*^), and we consider

(31) ∑k=1∞fkuk|xk+1|pk−∑k=1∞fkuk|Δr(kxk)|pk  ≤∑k=1∞fkukkpk|Δr(kxk)|pk.

Then the series ∑_*k*=1_^*∞*^ *f*_*k*_ *u*_*k*_ | *x*_*k*+1_|^*p*_*k*_^ is convergent from the definition of *l*_*p*_. Also, ∑_*k*=1_^*∞*^ *f*_*k*_ *u*_*k*_ | Δ^*r*^(*kx*_*k*_)|^*p*_*k*_^ < *∞*, and therefore *x* ∈ *l*_*p*_(*ℱ*, *u*, Δ^*r*^)∩∫*bv* ^*p*^(*ℱ*, *u*, Δ^*r*^).

Then,

(32) $$\begin{array}{c} h_{p}\left(ℱ,u,\Delta^{r}\right)\subset l_{p}\left(ℱ,u,\Delta^{r}\right)\cap \int bv^{p}\left(ℱ,u,\Delta^{r}\right). \end{array}$$

From (30) and (32), we have

(33) $$\begin{array}{c} h_{p}\left(ℱ,u,\Delta^{r}\right)=l_{p}\left(ℱ,u,\Delta^{r}\right)\cap \int bv^{p}\left(ℱ,u,\Delta^{r}\right). \end{array}$$

**Theorem 6 .**

The sequence space *h*_*p*_(*ℱ*, *u*, Δ^*r*^) is a BK-space with AK.

**Proof**

If *x* is any sequence, we write *σ*_*n*_(*x*) = *M*_*n*_ *x*. Let *ϵ* > 0 and *x* ∈ *h*_*p*_(*ℱ*, *u*, Δ^*r*^). Then, their exists *N* such that

(34) $$\begin{array}{c} \sigma_{n}\left(x\right)<\frac{\epsilon }{2} \end{array}$$

for all *n* ≥ *N*. Now let *m* ≥ *N* be given. Then, we have for all *n* ≥ *m* + 1 by (34)

(35) |σn(x−x[m])|≤[∑k=m+1∞fk(|kukΔrxk|pk)]1/pk≤|σn(x)|+|σm(x)|<ϵ2+ϵ2=ϵ,

whence ||*x*−*x* ^[*m*]^||_*h*_*p*_(*ℱ*,*u*,Δ^*r*^)_ ≤ *ϵ* for all *m* > *N*. This shows that *x* = lim⁡_*m*→*∞*_ *x* ^[*m*]^.

Since *h*_*p*_(*ℱ*, *u*, Δ^*r*^) is an* AK*-space and every* AK*-space is* AD*, we can give the following corollary.

**Corollary 7 .**

The sequence space *h*_*p*_(*ℱ*, *u*, Δ^*r*^) is AD.

## 3. Duals of Hahn Sequence Space *h*_*p*_(*ℱ*,  *u*,  Δ^*r*^)

In this section, we determining the *α*, *β*, and *γ*-duals of the sequence space *h*_*p*_(*ℱ*, *u*, Δ^*r*^). Let *x* and *y* be sequences, *X* and *Y* be subsets of *w*, and *A* = (*a*_*nk*_)_*n*,*k*=0_^*∞*^ be an infinite matrix of complex numbers. We write *xy* = (*x*_*k*_ *y*_*k*_)_*k*=0_^*∞*^, *x* ^−1^∗*Y* = {*a* ∈ *w* : *ax* ∈ *Y*}, and *M*(*X*, *Y*) = ∩_*x*∈*X*_ *x* ^−1^∗*Y* = {*a* ∈ *w* : *ax* ∈ *Y*  for  all  *x* ∈ *X*} for the multiplier space of *X* and *Y*. In the special cases of *Y* = {*l*_1_, *cs*, *bs*}, we write *x* ^*α*^ = *x* ^−1^∗*l*_1_, *x* ^*β*^ = *x* ^−1^∗*cs*, *x* ^*γ*^ = *x* ^−1^∗*bs* and *X* ^*α*^ = *M*(*X*, *l*_1_), *X* ^*β*^ = *M*(*X*, *cs*), *X* ^*γ*^ = *M*(*X*, *bs*) for the *α*-dual, *β*-dual, and *γ*-dual of *X*. By *A*_*n*_ = (*a*_*nk*_)_*k*=0_^*∞*^, we denote the sequence in the *n*th-row of *A*, and we write *A*_*n*_(*x*) = ∑_*k*=0_^*∞*^ *a*_*nk*_ *x*_*k*_ *n* = (0,1,…) and *A*(*x*) = (*A*_*n*_(*x*))_*n*=0_^*∞*^, provided that *A*_*n*_ ∈ *x* ^*β*^ for all *n*.

Given an *FK*-space *X* containing *ϕ*, its conjugate is denoted by *X*′ and its *f*-dual or sequential dual is denoted by *X* ^*f*^ and is given by *X* ^*f*^ = {all  sequences  (*f*(*e* ^*k*^)) : *f* ∈ *X*′}. Let *λ* be a sequence space. Then *λ* is called perfect if *λ* = *λ* ^*αα*^, normal if *y* ∈ *λ* whenever |*y*_*k*_ | ≤|*x*_*k*_ | , *k* ≥ 1 for some *x* ∈ *λ*, and monotone if *λ* contains the canonical preimages of all its stepspace.

**Lemma 8 .**

(i) *A* ∈ (*h* : *l*_1_) if and only if

(36) $$\begin{array}{c} \overset{\infty }{\underset{n=1}{\sum }}\left|a_{nk}\right|\;\;converges,\;\left(k=1,2,3,\ldots \right),\\ \underset{k}{\sup}\frac{1}{k}\overset{\infty }{\underset{n=1}{\sum }}\left|\overset{k}{\underset{v=1}{\sum }}a_{nv}\right|<\infty . \end{array}$$

(ii) *A* ∈ (*l*_*p*_ : *l*_1_) if and only if

(37) $$\begin{array}{c} \underset{K\in F}{\sup}\underset{k}{\sum }{\left|\underset{n\in K}{\sum }a_{nk}\right|}^{q}<\infty . \end{array}$$

**Lemma 9 .**

(i) *A* ∈ (*h* : *C*) if and only if

(38) $$\begin{array}{c} \underset{n,k}{\sup}\frac{1}{k}\left|\overset{k}{\underset{v=1}{\sum }}a_{nv}\right|<\infty , \end{array}$$

(39) $$\begin{array}{c} \underset{n\to \infty }{\lim}a_{nk}\;\;exists,\;\left(k=1,2,3,\ldots \right). \end{array}$$

(ii) *A* ∈ (*l*_*p*_ : *C*) if and only if (39) holds and

(40) $$\begin{array}{c} \underset{n}{\sup}\underset{k}{\sum }{\left|a_{nk}\right|}^{q}<\infty \;1<p\leq \infty . \end{array}$$

**Lemma 10 .**

(i) *A* ∈ (*h* : *l*_*∞*_) if and only if (38) holds.

(ii) *A* ∈ (*l*_*p*_ : *l*_*∞*_) if and only if (40) holds with 1 < *p* ≤ *∞*.

**Lemma 11 .**

(i) *A* ∈ (*h* : *C*_0_) if and only if (38) holds and

(41) $$\begin{array}{c} \underset{n\to \infty }{\lim}a_{nk}=0. \end{array}$$

**Lemma 12 .**

(i) *A* ∈ (*h* : *h*) if and only if (41) holds and

(42) $$\begin{array}{c} \overset{\infty }{\underset{n=1}{\sum }}n\left|a_{nk}-a_{n+1,k}\right|\;\;converges,\;\left(k=1,2,3,\ldots \right),\\ \underset{k}{\sup}\frac{1}{k}\overset{\infty }{\underset{n=1}{\sum }}n\left|\overset{k}{\underset{v=1}{\sum }}\left(a_{nv}-a_{n+1,v}\right)\right|<\infty . \end{array}$$

**Theorem 13 .**

We define the set

(43) d1={a=(ak)∈w:sup⁡K∈F∑k|∑n∈K1kfk(|ukΔrxk|)an|pk<∞}.

Then, [*h*_*p*_(*ℱ*, *u*, Δ^*r*^)]^*α*^ = *d*_1_.

**Proof**

Let us take any *a* = (*a*_*k*_) ∈ *w*. We define the matrix *D* = *d*_*nk*_ by

(44) $$\begin{array}{c} d_{nk}=\{\begin{array}{cc} \frac{1}{k}f_{k}\left(\left|u_{k}\Delta^{r}\right|\right)a_{n}, & k\geq n;\\ 0, & k<n \end{array} \end{array}$$

for all, *k*, *n* ∈ *ℕ*.

Consider the equation

(45) 1kfk(|ukΔr|)anxn=∑j=n∞1jfj(|ujΔr|)anyj=(Dy)n(n∈ℕ).

It follows from (45) with Lemma 8(ii) that *ax* = (*a*_*n*_ *x*_*n*_) ∈ *l*_1_ whenever *x* = (*x*_*k*_) ∈ *h*_*p*_(*ℱ*, *u*, Δ^*r*^) if and only if *Dy* ∈ *l*_1_, whenever *y* = (*y*_*k*_) ∈ *l*_*p*_. This means that *a* = (*a*_*n*_)∈[*h*_*p*_(*ℱ*, *u*, Δ^*r*^)]^*α*^, whenever *x* = (*x*_*k*_) ∈ *h*_*p*_(*ℱ*, *u*, Δ^*r*^) if and only if *D* ∈ (*h*_*p*_(*ℱ*, *u*, Δ^*r*^) : *l*_1_). This gives the result that [*h*_*p*_(*ℱ*, *u*, Δ^*r*^)]^*α*^ = *d*_1_.

**Theorem 14 .**

Let 1 < *p* < *∞*. Then [*h*_*p*_(*ℱ*, *u*, Δ^*r*^)]^*β*^ = *d*_2_, where

(46) d2={a=(ak)∈w:sup⁡n∈ℕ(n−1)q×∑kfk(|ukΔrxk|)|∑j=knaj|q<∞}.

**Proof**

Consider the equation

(47) ∑k=1n1kfk(|ukΔr|)akxk  =∑k=1n1kfk(|ukΔr|)ak(∑j=1kyjj)  =∑k=1n(∑j=1k1kfj(|ujΔr|)aj)yk  =(By)n (n∈ℕ),

where *B* = (*b*_*nk*_) are defined by

(48) $$\begin{array}{c} b_{nk}\{\begin{array}{cc} \overset{k}{\underset{j=1}{\sum }}\frac{1}{k}f_{j}\left(\left|u_{j}\Delta^{r}\right|\right)a_{j}, & k\geq n;\\ 0, & k<n \end{array} \end{array}$$

for all, *k*, *n* ∈ *ℕ*. Thus, we deduce from Lemma 9(ii) with (47) that *ax* = (*a*_*k*_ *x*_*k*_) ∈ *cs* whenever *x* = (*x*_*k*_) ∈ *h*_*p*_(*ℱ*, *u*, Δ^*r*^) if and only if *By* ∈ *c* whenever *y* = (*y*_*k*_) ∈ *l*_*p*_. Thus (*a*_*k*_) ∈ *cs* and (*a*_*k*_)*ind*_2_ by (39) and (40), respectively. Nevertheless, the inclusion *d*_2_ ⊂ *cs* holds, and thus, we have (*a*_*k*_) ∈ *d*_2_, whence [*h*_*p*_(*ℱ*, *u*, Δ^*r*^)]^*β*^ = *d*_2_.

**Lemma 15 .**

Let *X* be FK-space with *X*⊃*φ*. Then,

- *X* ^*β*^ ⊂ *X* ^*γ*^ ⊂ *X* ^*f*^;
- If *X* has AK, *X* ^*β*^ = *X* ^*f*^;
- If *X* has AD, *X* ^*β*^ = *X* ^*γ*^.

From Theorem 6, Corollary 7, and Lemma 15, we can write the following corollary.

**Corollary 16 .**

(i) [*h*_*p*_(*ℱ*, *u*, Δ^*r*^)]^*β*^ = [*h*_*p*_(*ℱ*, *u*, Δ^*r*^)]^*f*^;

(ii) [*h*_*p*_(*ℱ*, *u*, Δ^*r*^)]^*β*^ = [*h*_*p*_(*ℱ*, *u*, Δ^*r*^)]^*γ*^.

**Lemma 17 .**

Let *λ* be a sequence space. Then, the following assertions are true:

- *λ* is perfect ⇒*λ* is normal ⇒*λ* is monotone;
- *λ* is normal ⇒*λ* ^*α*^ = *λ* ^*γ*^;
- *λ* is monotone ⇒*λ* ^*α*^ = *λ* ^*β*^.

Combining Theorem 13, Theorem 14, and Lemma 17, we can give the following corollary.

**Corollary 18 .**

The space *h*_*p*_(*ℱ*, *u*, Δ^*r*^) is not monotone and so it is neither normal nor perfect.

## 4. Matrix Transformations

In this section we characterize some matrix transformations on the space *h*_*p*_(*ℱ*, *u*, Δ^*r*^).

**Lemma 19 .**

Let *λ*, *μ* be any two sequence spaces, *A* be an infinite matrix, and *U* be a triangle matrix. Then, *A* ∈ (*λ* : *μ*_*U*_) if and only if *UA* ∈ (*λ* : *U*).

If we define ${\tilde{a}}_{nk}=n(a_{nk}-a_{n+1,k})$, then we can give the following corollary from Lemma 19 with *U* = *M* defined by (16).

**Corollary 20 .**

(i) *A* ∈ (*l*_1_ : *h*) if and only if

(49) $$\begin{array}{c} \underset{k}{\sup}\;\underset{n}{\sum }\left|{\tilde{a}}_{nk}\right|<\infty . \end{array}$$

(ii) *A* ∈ (*c* : *h*) = (*c*_0_ : *h*) = (*l*_*∞*_ : *h*) if and only if

(50) $$\begin{array}{c} \underset{K\in F}{\sup}\;\underset{n}{\sum }\left|\underset{k\in K}{\sup}\;{\tilde{a}}_{nk}\right|<\infty . \end{array}$$

**Theorem 21 .**

Suppose that the entries of the infinite matrices *A* = (*a*_*nk*_) and *E* = (*e*_*nk*_) are connected with the relation

(51) $$\begin{array}{c} e_{nk}={\bar{a}}_{nk} \end{array}$$

for all *k*, *n* ∈ *ℕ*, where ${\bar{a}}_{nk}=\sum_{j=k}^{\infty }\left(a_{nj}/j\right)[f_{j}(|u_{j}\Delta^{r}x_{j}|)]$ and *μ* is any sequence space. Then *A* ∈ (*h*_*p*_(*ℱ*, *u*, Δ^*r*^) : *μ*) if and only if {*a*_*nk*_}_*k*∈*ℕ*_ ∈ [*h*_*p*_(*ℱ*, *u*, Δ^*r*^)]^*β*^, for all *n* ∈ *ℕ* and *E* ∈ (*h* : *μ*).

**Proof**

Let *μ* be any given sequence spaces. Suppose that (51) holds between *A* = (*a*_*nk*_) and *E* = (*e*_*nk*_), and take into account that the spaces *h*_*p*_(*ℱ*, *u*, Δ^*r*^) and *h* are norm isomorphic. Let *A* ∈ (*h*_*p*_(*ℱ*, *u*, Δ^*r*^) : *μ*) and take any *y* = *y*_*k*_ ∈ *h*. Then, *EM* exists and {*a*_*nk*_}_*k*∈*𝕂*_ ∈ [*h*_*p*_(*ℱ*, *u*, Δ^*r*^)]^*β*^, which yields that {*e*_*nk*_}_*k*∈*ℕ*_ for each *n* ∈ *ℕ*. Hence, *Ey* exists, and thus,

(52) $$\begin{array}{c} \underset{k}{\sum }f_{k}\left(\left|u_{k}\Delta^{r}\right|\right)e_{nk}y_{k}=\underset{k}{\sum }f_{k}\left(\left|u_{k}\Delta^{r}\right|\right)a_{nk}x_{k} \end{array}$$

for all *n* ∈ *ℕ*. We have *Ey* = *Ax* which leads us to the consequence *E* ∈ (*h* : *μ*). Conversely, let {*a*_*nk*_}_*k*∈*𝕂*_ ∈ *d*_1_ for all *n* ∈ *ℕ*, and *E* ∈ (*h* : *μ*) hold, and take any *x* = *x*_*k*_ ∈ *h*_*p*_(*ℱ*, *u*, Δ^*r*^). Then, *Ax* exists. Therefore, we obtain from the equality that

(53) $$\begin{array}{c} \underset{k}{\sum }f_{k}\left(\left|u_{k}\Delta^{r}\right|\right)a_{nk}x_{k}=\left[\overset{\infty }{\underset{j=k}{\sum }}\frac{a_{nj}}{j}\right]\underset{k}{\sum }f_{k}\left(\left|u_{k}\Delta^{r}\right|\right)y_{k} \end{array}$$

for all *n* ∈ *ℕ*. Thus, *Ax* = *Ey* and this shows that *A* ∈ (*h*_*p*_(*ℱ*, *u*, Δ^*r*^) : *μ*).

If we use the Corollary 20 and change the roles of the spaces *h*_*p*_(*ℱ*, *u*, Δ^*r*^) with *μ* in Theorem 21, we can give the following theorem.

**Theorem 22 .**

Suppose that the entries of the infinite matrices *A* = (*a*_*nk*_) and $\tilde{A}=({\tilde{a}}_{nk})$ are connected with the relation ${\tilde{a}}_{nk}=nf_{k}(|u_{k}\Delta^{r}x_{k}|)(a_{nk}-a_{n+1,k})$ for all *k*, *n* ∈ *ℕ* and *μ* is any sequence space. Then *A* ∈ (*μ* : *h*_*p*_(*ℱ*, *u*, Δ^*r*^)) if and only if $\tilde{A}\in (\mu :h)$.

**Proof**

Let *z* = *z*_*k*_ ∈ *μ* and consider the following equality:

(54) ∑k=0ma~nkzk=∑k=0mnfk(|ukΔr|)(ank−an+1,k)zk,∀m,n∈ℕ

which yields that as $m\to \infty \;{\left(\tilde{A}z\right)}_{n}={\left(M(Az)\right)}_{n}$ for all *n* ∈ *ℕ*. Therefore, one can observe from here that *Az* ∈ *h*_*p*_(*ℱ*, *u*, Δ^*r*^) whenever *z* ∈ *μ* if and only if $\tilde{A}z\in h$ whenever *z* ∈ *μ*.
